# Supplementary figures and images for: Epinephrine Affects Ribosomes, Cell Division, and Catabolic Processes in Micrococcus luteus Skin Strain C01: Revelation of the Conditionally Extensive Hormone Effect Using Orbitrap Mass Spectrometry and Proteomic Analysis
Source: Microorganisms. 2023 Aug 29;11(9):2181. doi: 10.3390/microorganisms11092181 (PMC10535722; doi:10.3390/microorganisms11092181)

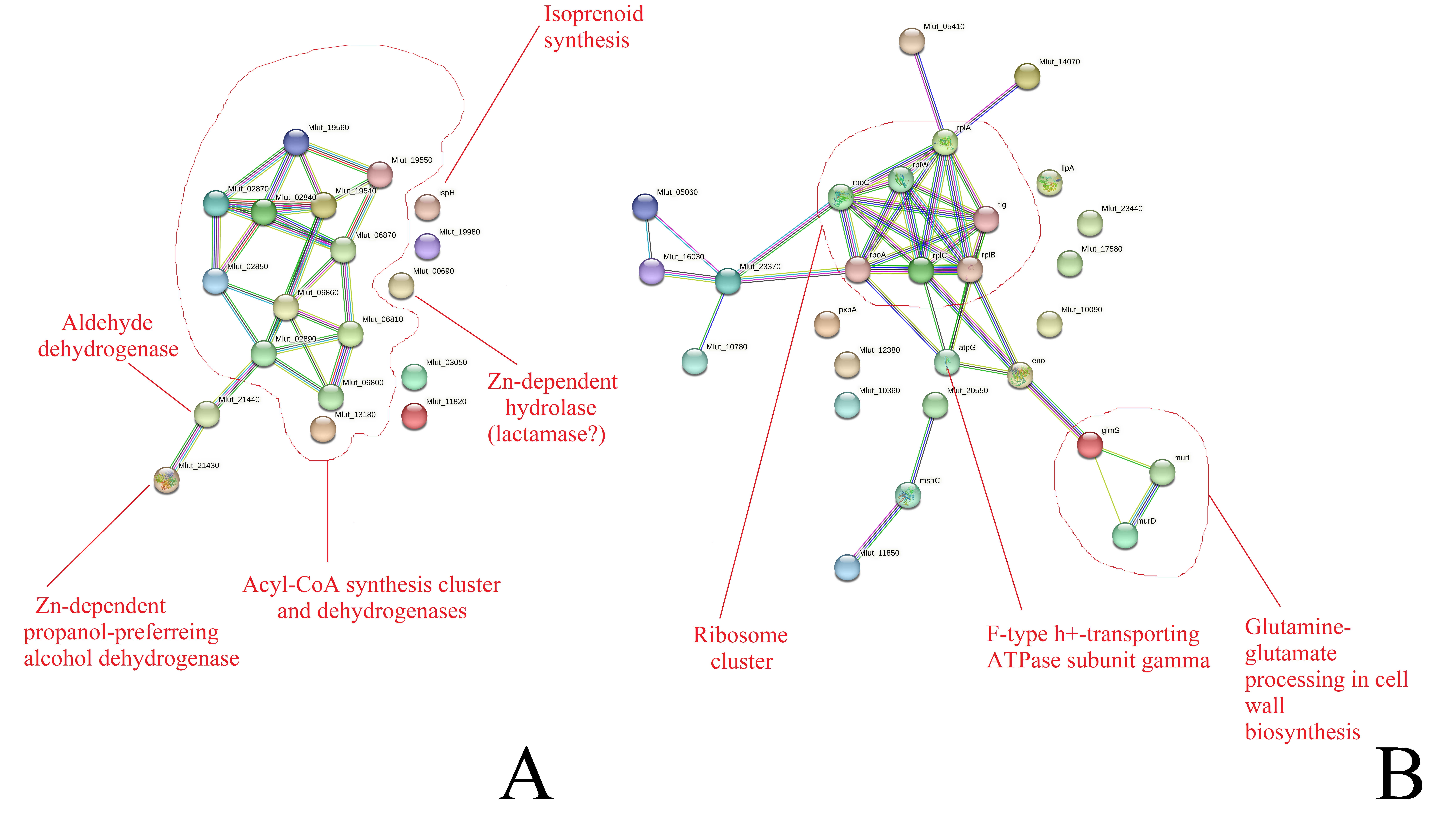

Supplement: Supplementary file 1 [file microorganisms-11-02181-s001.zip › Supplementary figures/Figure S1.png]
